# The Medical Remembrancer, or Book of Emergencies

**Published:** 1845-01

**Authors:** 


ART. X.—1. *The Medical Remembrancer, or Book of Emergencies; in which are concisely pointed out the immediate remedies to be adopted in the first moments of danger from Poisoning, Drowning, Apoplexy, Burns, and other Accidents; with the Tests for the principal Poisons, and other useful information.* By E. B. L. SHAW, Surgeon. Second Edition.—*London*, 1845. 18mo, pp. 108.

2. *The Prescriber's Pharmacopœia: containing all the Medicines in the London Pharmacopœia, arranged in classes according to their action, with their Composition and Doses.* By a Practising Physician. Third Edition.—*London*, 1845. 18mo, pp. 144.

1. THE plan of the first of these little books is well conceived, and the execution corresponds thereunto. Its principal contents are detailed in the title above transcribed. It costs little money, and will occupy little room; and we think no practitioner will regret being the possessor of what cannot fail, sooner or later, to be useful to him. The only criticism we have to offer, is on the part devoted to "the minor operations." These are clearly out of place in such a work as this. What have bleeding, cupping, electricity, issues, and vaccination, to do with sudden emergencies?

2. The second of our Lilliputian friends is an old acquaintance and old favorite; and we doubt not, from the additions which we see have been made to it, that, in its present form, "it will be found," as the editor hopes, "still more deserving the patronage of those for whom it is intended." It is truly the prescriber's book,—and we advise all who prescribe, to add it to their Christmas stock of purchases. They will thank us for advising them to do so. It is a medical Christmas-pie.

